# Supplementary material for: Comparative multiomics analysis of cell physiological state after culture in a basket bioreactor
Source: Sci Rep. 2022 Nov 23;12:20161. doi: 10.1038/s41598-022-24687-4 (PMC9686226; doi:10.1038/s41598-022-24687-4)

_023666047.1:3765163:3766608:+@NW_023666047.1:3765163:3767110:+@NW_023666047.1:3767007:37671[illegible]

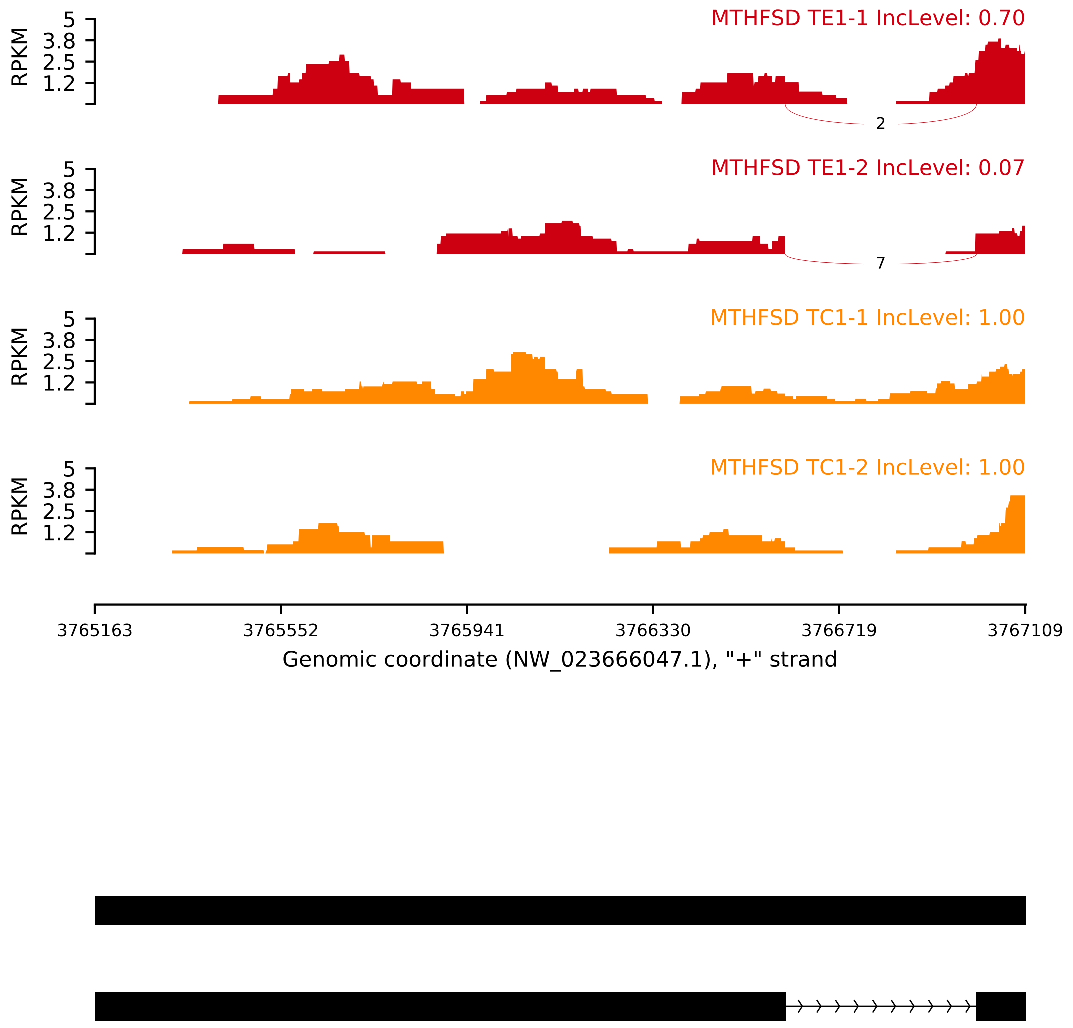

Supplement: Supplementary file 1 — Supplementary Information 1. [file 41598_2022_24687_MOESM1_ESM.zip › raw data/Transcriptomic raw data/7.AS/2.dasplot/TE1vsTC1/RI/13_MTHFSD_NW_023666047.1_3765163_3766608_+@NW_023666047.1_3765163_3767110_+@NW_023666047.1_3767007_3767110_+.pdf]
